# The CELL NUMBER REGULATOR SlFWL5 protein regulates aerial vegetative growth in tomato, by promoting cell expansion

**DOI:** 10.1093/jxb/eraf444

**Published:** 2025-10-15

**Authors:** Arthur Beauchet, Lucie Ehrhard, Lina Boutaleb, Valérie Rofidal, Nathalie Gonzalez, Christian Chevalier, Norbert Bollier

**Affiliations:** INRAE, Université de Bordeaux, UMR1332 Biologie du Fruit et Pathologie, Villenave d’Ornon 33140, France; INRAE, Université de Bordeaux, UMR1332 Biologie du Fruit et Pathologie, Villenave d’Ornon 33140, France; INRAE, Université de Bordeaux, UMR1332 Biologie du Fruit et Pathologie, Villenave d’Ornon 33140, France; IPSiM, CNRS, INRAE, Institut Sup Agro, Université de Montpellier, Montpellier 34060, France; INRAE, Université de Bordeaux, UMR1332 Biologie du Fruit et Pathologie, Villenave d’Ornon 33140, France; INRAE, Université de Bordeaux, UMR1332 Biologie du Fruit et Pathologie, Villenave d’Ornon 33140, France; INRAE, Université de Bordeaux, UMR1332 Biologie du Fruit et Pathologie, Villenave d’Ornon 33140, France; University of California, Davis, USA

**Keywords:** Cell expansion, CELL NUMBER REGULATOR, FW2.2-LIKE, hypocotyl growth, leaf growth, plasma membrane, plasmodesmata, tomato

## Abstract

The *FW2.2-LIKE/CELL NUMBER REGULATOR* (*FWL/CNR*) gene family comprises PLAC8 domain-containing membrane-associated proteins and was named in reference to its founding member, the *FW2.2* gene, which determines fruit size in tomato via a negative regulation on cell divisions. The function of PLAC8 domain-containing FWL/CNR proteins in plants remains largely unexplored. Only recently has the molecular and cellular mechanism of FW2.2 been described as regulating plasmodesmata-mediated cell-to-cell communication. In the present study, we provided a functional analysis of *SlFWL* genes in tomato, aiming at investigating any direct role in the control of organ growth. Based on a combination of molecular and cellular approaches, we selected three SlFWL proteins, namely SlFWL2, -4, and -5, which are localized at the plasma membrane. Gain- and loss-of-function transgenic plants were generated to explore their putative role as regulators of organ growth in tomato. This allowed us to shed light more specifically on the critical involvement of SlFWL5 in leaf and hypocotyl development. We show here that SlFWL5 is localized at plasmodesmata, and that it regulates leaf size and morphology, and hypocotyl growth, by promoting cell expansion—unexpected for a CELL NUMBER REGULATOR protein. This original finding underscores further the importance of some FWL/CNR family members in growth regulation.

## Teaser

The FW2.2-LIKE/CELL NUMBER REGULATOR protein SlFWL5 is localized at plasmodesmata and regulates aerial vegetative growth in tomato, via cell expansion.

## Introduction

The *FW2.2-LIKE/CELL NUMBER REGULATOR* (*FWL/CNR*) gene family (Guo *et al.*, 2010; Thibivilliers *et al.*, 2020) was named in reference to its founding member, the *FW2.2* gene (standing for Fruit Weight QTL on chromosome 2, number 2), which underlies the major quantitative trait locus (QTL) governing fruit size in tomato (Alpert *et al.*, 1995; Frary *et al.*, 2000). The *FWL/CNR* gene family encompasses hundreds of related sequences in the plant, animal, and fungal reigns (Guo *et al.*, 2010), with a large diversity in protein size ranging from a hundred to several hundred amino acids. The FWL/CNR proteins are usually described as membrane-associated Pro- and Cys-rich proteins, which harbor, as a principal feature, the presence of a conserved structural domain named PLAC8 (Placenta specific 8), originally identified in a protein specifically expressed in the trophoblast giant cells and spongiotrophoblast layer of the placenta from mouse (Galaviz-Hernandez *et al.*, 2003). The PLAC8 domain is composed of one or two hydrophobic segments, predicted to form two putative transmembrane (TM) α-helices, and characterized by the presence of Cys-rich motifs of the CLXXXXCPC or CCXXXXCPC type (Song *et al.*, 2004; Beauchet *et al.*, 2021).

Three main functions have been assigned to PLAC8 domain-containing FWL/CNR proteins in plants: the regulation of organ size, especially in fruits; cadmium resistance; and metal ion homeostasis (Beauchet *et al.*, 2021). Indeed, the original FW2.2 protein negatively regulates cell divisions in the pre-anthesis ovary and young developing fruit, thereby accounting for its quantitative effects on final fruit size via the modulation of cell number (Nesbitt and Tanksley, 2001; Cong *et al.*, 2002; Liu *et al.*, 2003). Functional analyses of FWL/CNR proteins suggest that this role in the regulation of organ growth, primarily through their effects on cell division, is a highly conserved feature in both monocotyledon and dicotyledon plants (Dahan *et al.*, 2010; Guo *et al.*, 2010; Li and He, 2015; Qiao *et al.*, 2017; Ruan *et al.*, 2020). Interestingly, and related to this function, the PLAC8 protein has also been determined to be involved in organ development and cell division control during tumorigenesis in mammals (Cabreira-Cagliari *et al.*, 2018; Mao *et al.*, 2021). However, the precise molecular mechanism underlying the role of FWL/CNR proteins in regulating organ growth linked to cell division/cell cycle control remains largely unexplored.

Recently, Beauchet *et al.* (2024) made a major breakthrough towards the identification of the molecular and cellular mode of action of FW2.2 in tomato. In this study, FW2.2 was demonstrated to be a membrane-anchored protein that locates at plasmodesmata (PDs) and regulates cell-to-cell communication by modulating PD transport capacity during fruit development, via the regulation of callose deposition at PDs (Beauchet *et al.*, 2024). Whether this function is conserved in FWL/CNR proteins from other plant species remains to be determined. Nevertheless, several clues seem to comfirm this functionality. Indeed, the closest ortholog of FW2.2 in Arabidopsis, namely AtPCR2, is highly enriched at PDs (Brault *et al.*, 2019). The soybean GmFWL1 and GmFWL3 proteins are localized to membrane microdomains, and interact with structural proteins of the membrane microdomains, but also with proteins involved in the metabolic process of callose deposition, such as callose synthases (Qiao *et al.*, 2017; Cervantes-Pérez *et al*., 2024), like FW2.2 in tomato fruit (Beauchet *et al.*, 2024).

An updated phylogenetic analysis performed on 13 different plant genomes released in recent years allowed the identification of 134 members in the FWL/CNR family, with a total number of 12 *FWL* genes including *FW2.2* in tomato (Thibivilliers *et al.*, 2020). This list has been recently extended up to 21 members (Ran *et al.*, 2023), providing a valuable resource for the characterization of *FWL* genes in tomato.

In the present study, we provide the first functional analysis of *SlFWL* genes in tomato. We aimed at investigating whether SlFWLs can play a direct role in the control of organ growth. First, we re-investigated the expression profiles of *SlFWL* genes in different organs of tomato plants and during fruit development, and analyzed their subcellular localization. Based on this combination of molecular and cellular approaches, we selected three *SlFWL* genes, namely *SlFWL2*, -*4*, and -*5*, and generated gain- and loss-of-function transgenic plants to explore their putative role as regulators of organ growth and development in tomato. This allowed us to shed light more specifically on the critical involvement of SlFWL5 in the regulation of cell expansion during leaf development and hypocotyl growth, further underscoring the importance of some FWL/CNR family members in growth regulation.

## Materials and methods

### Plant materials and growth conditions

Tomato [*Solanum lycopersicum* cv. Ailsa Craig (AC)] and tobacco (*Nicotiana benthamiana*) plants were grown in soil in a greenhouse under the following conditions: 16 h day/8 h night cycle, using a set of 100 W warm white LED projectors providing an irradiance of 100 μmol m^−2^ s^−1^ at the level of the canopy. The light spectrum was constituted by equivalent levels of blue irradiation (range 430–450 nm) and red irradiation (640–660 nm). For *in vitro* culture, tomato seeds were sterilized for 10 min under agitation in a solution of 3.2% bleach. Seeds were then washed three times with sterile water and dried under a laminar flow hood. Seeds were sown in Murashige and Skoog medium (1/4 MS) and transferred in a growth chamber under the following conditions: 16 h day/8 h night cycle, 22 °C/20 °C day/night, using white light (Osram L36 W/77 Fluora 1400 Im) providing 80–100 μmol m^−2^ s^−1^ intensity light at the stirring plate.

### Phylogenetic analysis and tools for the prediction of the CNR/FWL structure and topology

The 21 SlFWL protein sequences from tomato were mined from Thibivilliers *et al*. (2020) and Ran *et al*. (2023), and aligned using MEGA11 Muscle alignment (Tamura *et al*., 2021). Values for 1000 bootstrap replicates are shown on each branch.

DeepTMHMM (https://dtu.biolib.com/DeepTMHMM) (Hallgren *et al*., 2022, Preprint) was used to predict the presence of a TM helix in SlFWL proteins. The three-dimensional structure of the full-length SlFWL2, -3, and -5 proteins was obtained using Colabfold (Mirdita *et al*., 2022) The PPM 3.0 Web Server (https://opm.phar.umich.edu/ppm_server3_cgopm) (Lomize *et al*., 2022) was used with default parameters and plasma membrane (PM; plants) type to predict the topology and insertion of SlFWL2, -3, and -5 in the PM.

### Vector constructs and plant transformation

Vectors for the overexpression of *SlFWL* genes in plants were generated using the Gateway^®^ cloning system (Invitrogen, Carlsbad, CA, USA), following the manufacturer’s instruction. The *FWL* full-length coding sequence was amplified from cDNAs prepared from various tomato tissues (cv. AC) using PrimeSTAR MAX DNA polymerase (TAKARA BIO Inc., Kusatsu, Japan) and primers including the attB sites (Supplementary Table S1). The resulting PCR products were cloned into the corresponding Gateway vectors described in Supplementary Table S2. For CRISPR/Cas9 [clustered regularly interspaced palindromic repeats (CRISPR)/CRISPR-associated protein 9] mutagenesis, constructs were assembled using the Golden Gate cloning method according to a previous method (Beauchet *et al.*, 2024). Transgenic plants were generated by *Agrobacterium tumefaciens*- (strain C58C1) mediated transformation using explants of tomato cotyledons as described (Swinnen *et al.*, 2022).

### RNA extraction and quantitative reverse transcription–PCR analysis

Total RNA was isolated from cotyledons, hypocotyls, shoot apical meristems, leaves, roots, flowers, and pericarp tissues from fruits harvested at different developmental stages (5, 10, and 15 d post-anthesis), using TRIzol reagent (Invitrogen) in combination with the RNeasy Plant Mini Kit (Qiagen) following the manufacturer’s instructions. RNase-free DNase (Qiagen) treatment was performed on each sample. Reverse transcription was performed using the iScript™ cDNA Synthesis Kit (Bio-Rad, Hercules, CA, USA). Real-time PCR was performed using Gotaq^®^ qPCR mastermix (Promega, Madison, WI, USA) and a CFX 96 real-time system (Bio-Rad). Quantitative PCR (qPCR) primers were designed with PerlPrimer software (Marshall, 2004) to overlap two exons in order to limit genomic DNA amplification (Supplementary Table S2) and amplify a 80–200 bp long amplicon, with a *T*_m_ of 60 °C. The transcript levels of the expressed genes were normalized to that of the housekeeping genes: *SlTUBULIN* (Solyc04g081490) in combination with *SlNUDK* (Solyc01g089970) for fruit samples, or with *SlEIF4α* (Solyc12g095990) for other tissue samples.

### Subcellular localization analysis of SlFWL proteins

The subcellular localization of SlFWLs fused to yellow fluorescent protein (YFP) was observed using confocal imaging performed on a Zeiss LSM 880 confocal laser scanning microscope equipped with fast AiryScan, using a Zeiss Plan apochromat ×10 (NA 0.45) or a C PL APO ×63 (NA 1.4) oil-immersion objective.

The top three leaves of three independent *N. benthamiana* plants (6–8 weeks old) were agro-infiltrated with *35S::SlFWLs-YFP* constructs and plasmolyzed using 0.4 M sorbitol for 15 min before observation. Co-infiltrations with SP-mTagBFP2-HDEL as a marker of endoplasmic reticulum (ER) and mScarlet-l as a marker of both cytosol and nucleus localization were performed to ascertain the localization pattern. Staining with FM4.64 at a final concentration of 4 µM was used as a control for PM localization (Bolte *et al*., 2004). For FM4.64 imaging, excitation was performed at 561 nm and fluorescence emission was collected at 630–690 nm. For YFP imaging, excitation was performed at 514 nm and fluorescence emission collected at 520–580 nm. For mTagBFP2 imaging, excitation was performed at 405 nm and fluorescence emission was collected at 440–490 nm. For mScarlet-I imaging, excitation was performed at 561 nm and fluorescence emission was collected at 570–640 nm.

The localization of SlFWL2, -4, and -5 at the PM and PDs was performed in leaves of tomato *35S::SlFWL2-YFP*, *35S::SlFWL4-YFP*, and *35S::SlFWL5-YFP* transgenic plants. Similarly, leaf epidermal cells were plasmolyzed using 0.4 M sorbitol prior to observation. Staining with aniline blue (Biosupplies, Victoria, Australia) was performed by infiltration of a 0.0125% solution; excitation was performed at 405 nm and fluorescence emission collected at 420–480 .0nm. The PD index was determined by calculating the fluorescence intensity of SlFWL2–YFP, SlFWL4–YFP, and SlFWL5–YFP at PDs, and at the PM as described previously (Grison *et al*., 2019).

### Phenotypic characterization

Plants were cultivated randomly side-by-side with wild-type (WT) plants. Flowers were vibrated every day to ensure optimal self-pollination. Seven flowers per inflorescence were maintained to ensure proper development of fruit per inflorescence. Fruits from 4–6 plants of each genotype of two biological replicates were used to determine fruit weight at the breaker stage of fruit development. Fruits were weighted and measured using a caliper. The number of measurements ranged from *n*=50 to *n*=200 depending on the number of fruits produced by the different transgenic plants. For root and hypocotyl length measurements, seedlings were grown on 1/4 MS in 245×245×20 mm Nunc tissue culture plates placed vertically. Pictures of seedlings were digitized using a scanner every day, and root and hypocotyl length were measured using ImageJ software. For leaf surface phenotyping, pictures of full-grown leaves were taken using a Nikon D5300 and analyzed by intensity threshold filtering in ImageJ. For the analysis of leaf epidermal cell size, leaflets were cleared with 70% ethanol for several days before staining with 0.1% (w/v) Calcofluor white M2R stain for 48 h. Leaflet fragments were observed with a Zeiss Axioimager epifluorescence microscope equipped with a ×20 (NA 0.5) lens using a DAPI-BP filter and a Cool Snap HQ2 CCD Camera. For the measurements of hypocotyl epidermal cell size, freshly harvested hypocotyls were fixed in 4% (w/v) paraformaldehyde in 1× phosphate-buffered saline (PBS) under vacuum for 30 min, rinsed three times with 1× PBS, and incubated in Clearsee solution for at least 2 weeks. Samples were then stained with 0.1% (w/v) Calcofluor white M2R for 48 h in fresh Clearsee. Central epidermal cells of each hypocotyls were imaged with a Zeiss LSM 880 confocal microscope using a Plan apochromat ×10 dry objective (NA 0.45); excitation was performed at 405 nm (0.2% laser power) and fluorescence emission was collected between 430 nm and 490 nm.

### Immunolabeling of callose

The level of callose deposition was quantified in leaves from 4-week-old plants, by immunofluorescence labeling using a callose-specific antibody, as described in Beauchet *et al*. (2024). The signal intensity was subsequently quantified as a proxy of callose deposition at PDs, by integrating the gray value of all the pixels above the same threshold. A minimum of six measurements was performed at least on five sections from at least three different leaves from different plants, and the experiment was repeated twice.

### Co-immunoprecipitation and mass spectrometry analysis

Total protein extracts from 100 mg of *35S::SlFWL5-YFP* leaf tissue were prepared using the following buffer: 1× PBS, cOmplete Protease Inhibitor Cocktail tablets (Roche, Mannheim, Germany), and 1% Triton X-100. Samples were incubated in the extraction buffer at 4 °C for 30 min with agitation, and then centrifuged (16 000 *g*, 10 min, 4 °C). Prior to co-immunoprecipitation, western blotting was used to check the presence of the expressed tagged-SlFWL5–YFP protein in the supernatant. The supernatant containing the resuspended proteins was then used for immunoprecipitation assay using anti-Green Fluorescent Protein (GFP) microbeads provided in the μMACS Epitope Tag Protein Isolation Kit according to the manufacturer’s protocol (Miltenyi Biotec, Bergisch Gladbach, Germany). Protein concentration was determined by Bradford assay according to the manufacturer’s instructions [Pierce Coomassie Plus (Bradford) Assay kit; Thermo Scientific]. Approximately, 500 μg of soluble proteins were loaded for each co-immunoprecipitation assay. The LC-MS-MS experiment was carried out as reported previously (Beauchet *et al*., 2024).

Gene Ontology (GO) enrichment analysis was carried out on PLAZA 5.0 (Van Bel *et al*., 2022) using the Plaza workbench, with a significance threshold of 0.05 and without any data filter.

The MS proteomics data have been deposited to the ProteomeXchange Consortium via the PRIDE (Perez-Riverol *et al*., 2022) partner repository with the dataset identifier PXD061438.

## Results

### Phylogenetic classification and gene expression profiles of *SlFWL* genes

A recent genome-wide analysis of the *SlFWL* family identified a total of 20 *FW2.2* homologous sequences in tomato (Ran *et al.*, 2023). These authors named the different genes according to their location on the tomato chromosomes, not taking into account the anteriority of the *SlFWL* gene classification (although incomplete) proposed by Thibivilliers *et al.* (2020). To avoid additional confusion and to conform to the principle of anteriority, we proposed to keep the latter gene annotation and to assign ascending gene names to the nine new identified SlFWL sequences according to their phylogenetic relationship to FW2.2 (Table 1).

**Table 1.** Proposed nomenclature of *Solanum lycopersicum* FWL genes

Gene ID	Proposed name	Name from Thibivilliers *et al*. (2020)	Name from Ran *et al*. (2023)
Solyc02g090730	*FW2.2*	*FW2.2*	*SlFW2.2*
Solyc05g009620	*SlFWL1*	*SlFWL1*	*SlFWL9*
Solyc01g005470	*SlFWL2*	*SlFWL2*	*SlFWL1*
Solyc04g007900	*SlFWL3*	*SlFWL3*	*SlFWL8*
Solyc03g119660	*SlFWL4*	*SlFWL4*	*SlFWL6*
Solyc06g066590	*SlFWL5*	*SlFWL5*	*SlFWL13*
Solyc03g120600	*SlFWL6*	*SlFWL6*	*SlFWL7*
Solyc08g013910	*SlFWL7*	*SlFWL7*	*SlFWL14*
Solyc08g013920	*SlFWL8*	*SlFWL8*	*SlFWL15*
Solyc10g081410	*SlFWL9*	*SlFWL9*	*SlFWL18*
Solyc12g013570	*SlFWL10*	*SlFWL10*	*SlFWL20*
Solyc12g037950	*SlFWL11*	*SlFWL11*	*SlFWL21*
Solyc02g083540	*SlFWL12*		*SlFWL3*
Solyc10g084260	*SlFWL13*		*SlFWL19*
Solyc09g007490	*SlFWL14*		*SlFWL16*
Solyc10g018920	*SlFWL15*		*SlFWL17*
Solyc05g051690	*SlFWL16*		*SlFWL10*
Solyc03g093200	*SlFWL17*		*SlFWL5*
Solyc02g079390	*SlFWL18*		*SlFWL2*
Solyc06g048790	*SlFWL19*		*SlFWL11*
Solyc06g048810	*SlFWL20*		*SlFWL12*

**Fig. 1.**
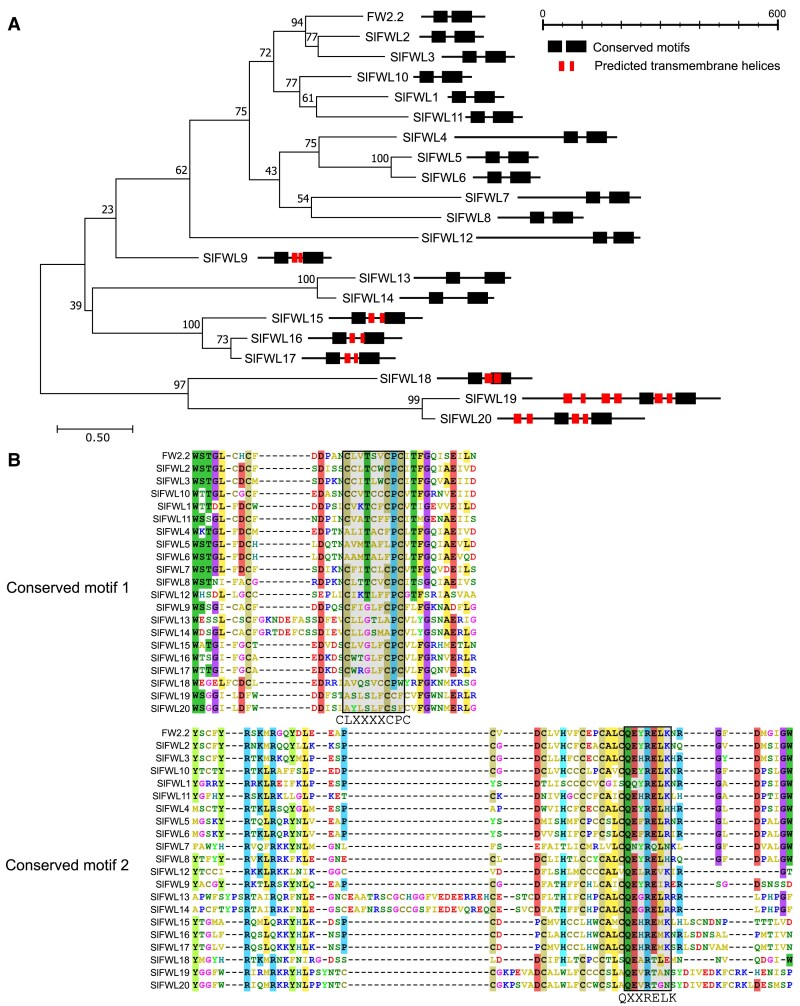
Phylogenetic analysis and protein structure of SlFWLs. (A) Phylogenetic tree built using the maximum likelihood method was generated using MEGA11 (Tamura *et al*., 2021). Values for 1000 bootstrap replicates are shown on each branch. The schematic representation of the protein structure of the SlFWL was deduced from computation analysis of protein primary sequences. Transmembrane domains were predicted using DeepTMHMM (https://dtu.biolib.com/DeepTMHMM) (Hallgren *et al*., 2022, Preprint). (B) Amino acid sequence comparison of the PLAC8 domain between SlFWLs and FW2.2, using Muscle alignment (MEGA11).

A phylogenic tree was thus generated using the full-length sequences of FW2.2 and the 20 homologous sequences from tomato (Fig. 1A). The SlFWLs fall into two distant clades: the first clade includes FW2.2 and the 11 sequences identified by Thibivilliers *et al.* (2020), with the addition of SlFWL12; the second clade encompasses SlFWL13–SlFWL20. Remarkably, SlFWLs from the first clade display a highly similar protein homology, with the exception of SlFWL4, SlFWL7, SlFWL8, and SlFWL12 which harbor a long N-terminal extension upstream of the conserved PLAC8 domain. SlFWLs from the second clade differ from the first one by a longer sequence, a greater distance between the two conserved motifs of the PLAC8 domain, and/or the presence of two or more predicted TM α-helices as revealed by the use of the TM topology prediction tool DeepTMHMM (Hallgren *et al.*, 2022, Preprint) (Fig. 1A). It is noteworthy that SlFWL9 occupies an intermediate position between the two subgroups, with a relatively shorter sequence than that of FW2.2 and the presence of two TM domains.

The primary structure of the conserved PLAC8 domains in the 20 SlFWL proteins is described in Fig. 1B. The CLXXXXCPC motif is only conserved in FW2.2, SlFWL8, and SlFWL15, while SlFWL13 and SlFWL14 harbor a CLXXXXAPC sequence, and SlFWL2, -3, and -10 harbor the CCXXXXCPC sequence. The CLXXXXCPC or CCXXXXCPC motif diverges more or less in the other SlFWL proteins, especially for the two first amino acids of the sequence. The second motif at the C-terminal part, the so-called ‘QXXRELK’ motif, appears much more conserved among the 20 SlFWL proteins.

To provide an overview of the expression pattern in tomato, the levels of transcript accumulation for *SlFWL* genes were monitored by quantitative reverse transcription–PCR (RT–qPCR) in various organs and fruit developmental stages from the tomato cultivar AC (Supplementary Fig. S1). This analysis showed that a large diversity of expression profiles was observed among the tested *SlFWL* genes, not only in terms of organs, but also in terms of the level of transcript accumulation. For instance, *SlFWL3*, *SlFWL14*, and *SlFWL15* exhibited much higher expression levels, ∼100-fold more, than all the other *SlFWL* genes. Most of the *SlFWL* genes are expressed to various levels throughout fruit development. *SlFWL3*, *SlFWL9*, *SlFWL10*, and *SlFWL15* were predominantly expressed in fruits, with *SlFWL3* and *SlFWL15* having the highest expression. *FW2.2*, *SlFWL1*, and *SlFWL4* were highly expressed in roots and to a lesser extent in fruit. *SlFWL2* was predominantly expressed in cotyledons, and to a lesser extent in hypocotyls, leaves, and flowers, and slightly in roots. *SlFWL5* showed high expression in both roots and cotyledons, and to a lower extent in leaves and hypocotyls. *SlFWL7* and *SlFWL8* were more specifically expressed in flowers. *SlFWL13*, *SlFWL14*, *SlFWL16*, *SlFWL17*, and *SlFWL18* were broadly expressed across nearly all tissues.

### Subcellular localization of SlFWL proteins

**Fig. 2.**
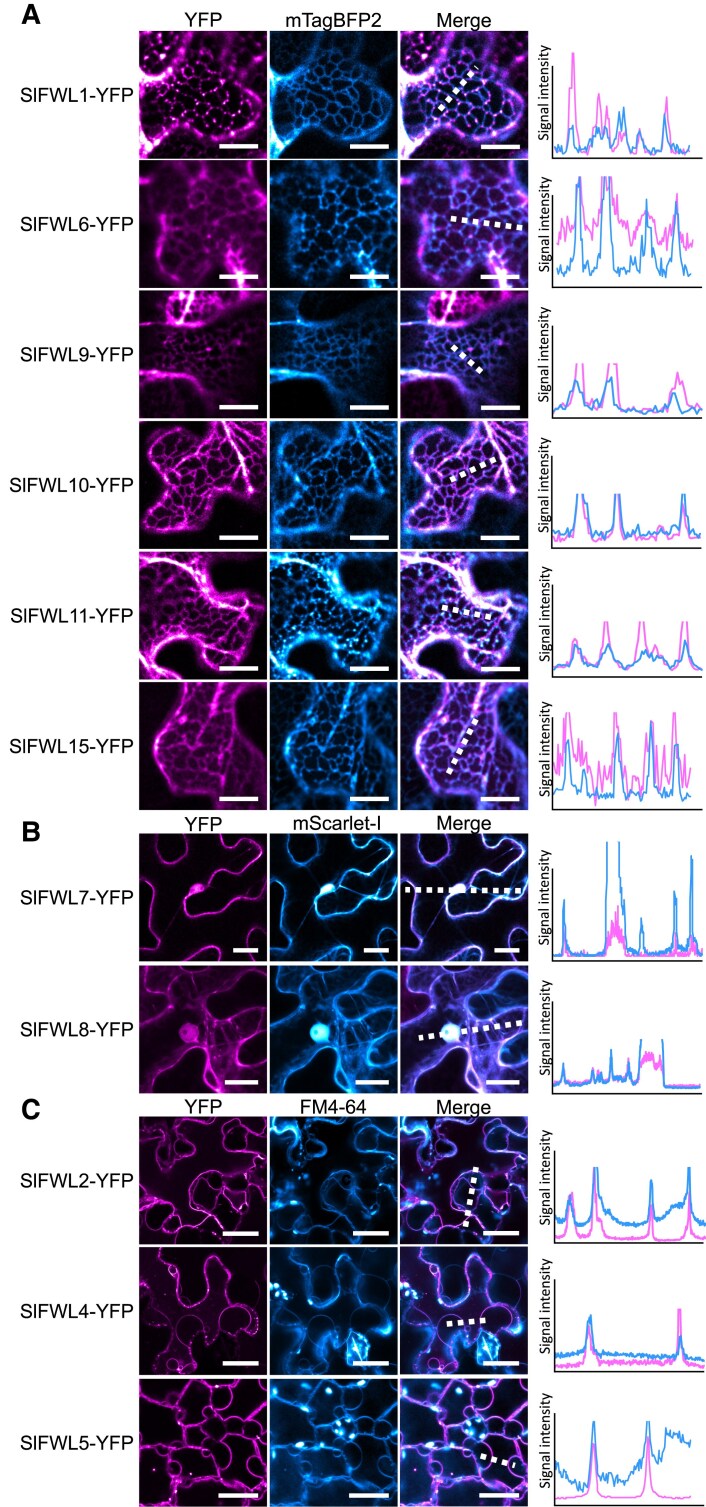
Subcellular localization of SlFWLs fused to YFP in *N. benthamiana* leaf epidermal cells. (A) ER localization revealed by the use of the ER-specific marker construct *pUBI::mTagBFP2-HDEL*. Scale bar=10 µm. (B) Cytosol and nuclear localization revealed by the use of the marker construct *pUBI::mScarlet-I*. Scale bar=10 µm. (C) PM localization revealed by the membrane-specific dye FM4-64. Scale bar=20 µm. Intensity plots delineated by the dashed lines are shown for each co-localization pattern.

The subcellular localization of SlFWL proteins was first investigated in *N. benthamiana* leaves, using transient expression assays. Constructs aimed at expressing the SlFWL proteins fused to YFP at the C-terminal end, under the control of the cauliflower mosaic virus (CaMV) 35S promoter, were thus generated (referred to as *35S::SlFWLX-YFP*). This type of construct was chosen based on our previous work, showing that the subcellular localization of FW2.2 was independent o the position of YFP at the C- or N-terminal end of the protein (Beauchet *et al.*, 2024). The data obtained for 11 out of 20 SlFWLs are displayed in Fig. 2. The following SlFWLs were not included in this analysis for the following reasons: (i) we could not determine the subcellular localization of SlFWL3, due to a lack of reproducibility in the results; (ii) we were unable to generate the constructs fusing the coding sequence for SlFWL12, -13, -14, -16, and -17 to YFP for cloning technical reasons; (iii) SlFWL18, -19, and -20 were excluded from the study because of their too distant phylogenetic relationship with the other SlFWLs.

For the tested SlFWLs, three distinct patterns of subcellular localization were observed. First, SlFWL1, -6, -9, -10, -11, and -15 were distributed within the cell in a reticular pattern, consistent with a localization at the ER. This ER localization was confirmed through co-infiltration with the ER marker SP-mTagBFP2-HDEL (kindly provided by Dr J. Dragdwidge, Ghent University, Belgium) and subsequent co-localization analysis (Fig. 2A).

Second, SlFWL7 and -8 were found to localize to both the cytosol and the nucleus. The use of the co-infiltrated mScarlet-l marker, a monomeric constitutive red fluorescent protein (Bindels *et al.*, 2017), confirmed this localization (Fig. 2B).

Third, SlFWL2, SlFWL4, and SlFWL5 were targeted to the PM, as witnessed by staining with FM4.64, a membrane-specific dye (Bolte *et al.*, 2004), following plasmolysis induced by a 0.4 M sorbitol treatment (Fig. 2C).

We next investigated the topology of these three SlFWLs at the PM using currently available prediction tools. First, the 3D structure of these PM-localized SlFWLs was predicted using ColabFold software (Mirdita *et al*., 2022) based on AlphaFold 2.0 (Jumper *et al*., 2021) and MMseqs2 (Steinegger and Söding, 2017). Second, the insertion in the PM was modelized using the PPM 3.0 Web Server (Lomize *et al*., 2022) (Supplementary Fig. S2). These predictions revealed that the 3D structures of SlFWL2, SlFWL4, and SlFWL5 present similarities to that from FW2.2 (Beauchet *et al.*, 2024), such as the absence of any TM domain and their N- and C-terminal parts predicted to be folded on the same side of the protein. These predictions suggest that they do not cross the PM, but are most probably anchored in the PM.

### *In planta* subcellular localization of SlFWL2, SlFWL4, and SlFWL5

Owing to their localization at the PM, we performed an *in planta* analysis of the subcellular localization of SlFWL2, SlFWL4, and SlFWL5, aimed at investigating whether these three SlFWLs are also enriched at PDs in tomato, like FW2.2 (Beauchet *et al.*, 2024). For this purpose, we generated stable transgenic lines expressing SlFWL2, SlFWL4, and SlFWL5 fused to YFP at the C-terminal end, under the control of the 35S promoter in the tomato cultivar AC (lines referred to as *35S::SlFWL2-YFP*, *35S::SlFWL4-YFP*, and *35S::SlFWL5-YFP* plants).

**Fig. 3.**
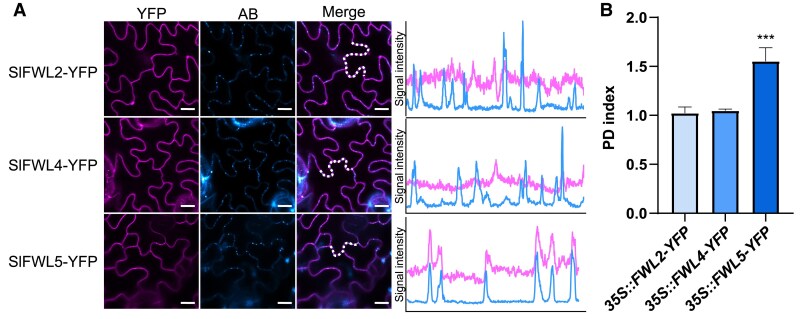
SlFWL5 is enriched at PDs. (A) Confocal microscope observations of SlFWL2, SlFWL4, and SlFWL5 localization in tomato leaf cells from *35S::SlFWL2-YFP*, *35S::SlFWL4-YFP*, and *35S::SlFWL5-YFP* plants. Scale bar=10 μm. Intensity plots delineated by the dashed lines are shown for each co-localization pattern. AB, aniline blue. (B) PD index for SlFWL2, SlFWL4, and SlFWL5 localization in leaf cells from *35S::SlFWL2-YF*P, *35S::SlFWL4-YFP*, and *35S::SlFWL5-YFP* plants. *n*>20 regions of interest from five images. Statistical analysis: Kruskal–Wallis test with post-hoc Dunn multiple comparison test. ***P<0.01.

The localization of SlFWL2–YFP, SlFWL4–YFP, and SlFWL5–YFP at the PM of tomato leaf cells was confirmed (Fig. 3A). In addition, SlFWL5–YFP was found to localize at the PM according to the pattern of punctate spots at the cell periphery, suggesting that SlFWL5–YFP was enriched at nanodomains. Staining with aniline blue to reveal callose deposition as a marker of PDs revealed that only SlFWL5–YFP co-localized with aniline blue, as shown by the overlapping signal intensity plots (Fig. 3A), thus indicating a localization at PDs. Next, we determined the PD enrichment ratio, named the ‘PD index’, which corresponds to the fluorescence intensity of SlFWL–YFP fusions at PDs versus that at the cell periphery, to quantify the enrichment of SlFWL–YFP fusions at PDs, as previously described (Beauchet *et al.*, 2024) (Fig. 3B). While the PD index for SlFWL2–YFP and SlFWL4–YFP was equal to 1, a high PD index from 1.5 to 1.7 was measured in leaf cells of *35S::SlFWL5-YFP* plants, thus demonstrating that only SlFWL5 out of the three SlFWLs tested was enriched at PDs.

### *In planta* functional analysis of *SlFWL2*, *SlFWL4*, and *SlFWL5*

To analyze the function of *SlFWL2*, *SlFWL4,* and *SlFWL5* in vegetative and reproductive development, gain- and loss-of-function plants were generated in the tomato cultivar AC. *SlFWL2*, *SlFWL4*, and *SlFWL5* were overexpressed constitutively and ectopically, using the 35S promoter (gain-of-function plants referred to as *35S::SlFWL2*, *-4*, and *-5* respectively). For each gene, three T_2_ lines were selected with medium to very high levels of *SlFWL2*, *-4*, and *-5* overexpression in leaves, ranging from 3-fold more to 150-fold more (Supplementary Fig. S3). Alternatively, *SlFWL2*, *SlFWL4,* and *SlFWL5* were knocked out using CRISPR/Cas9 technology. Two single guide RNAs (sgRNAs) per gene were designed close to the start codon, in order to create a frameshift or an early stop codon that would result in a dysfunctional protein (Supplementary Fig. S4). For each gene, we selected three independent T_0_ transgenic lines displaying homozygous mutations, either deletions or insertions, for subsequent phenotyping of vegetative and reproductive development. These loss-of-function plants are referred to as *CR-Slfwl2*, *-4*, and *-5* hereafter.

**Fig. 4.**
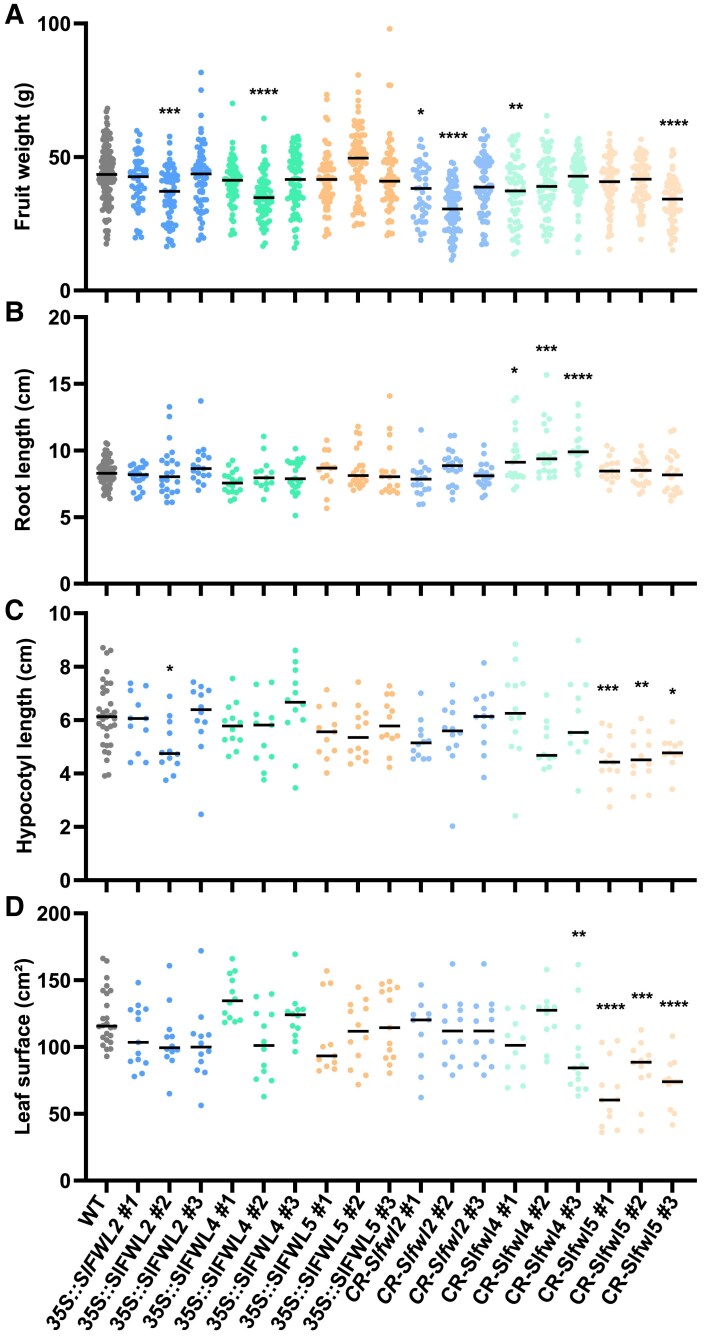
Phenotypic analysis of SlFWL2, SlFWL4, and SlFWL5 gain-of-function (referred to as *35S::SlFWL2*, *35S::SlFWL4*, and *35S::SlFWL5*) and loss-of-function (referred to as *CR-Slfwl2*, *CR-Slfwl4*, and *CR-Slfwl5*) plants compared with WT plants. (A) Fruit weight (at red ripe stage); *n*>40 fruits from four plants per line. (B) Root length (at 17 DAG); *n*>10 roots per line. (C) Hypocotyl length (at 17 DAG); *n*>10 hypocotyls per line. (D) Mature leaf surface; *n*>10 leaves from four plants per line. Statistical analysis: Kruskal–Wallis test with post-hoc Dunn multiple comparison test. *P<0.05; **P<0.01; ***P<0.001; ****P<0.0001.

The phenotypic analysis of the different transgenic plants is shown in Fig. 4. The overexpression of *SlFWL2*, *SlFWL4*, and *SlFWL5* did not induce any significant effects on either reproductive or vegetative development. Only one line each out of three for the *35S::SlFWL2* and *35S::SlFWL4* constructs, namely *35S::SlFWL2#2* and *35S::SlFWL4#2*, produced smaller fruits (−20% when compared with the WT) (Fig. 4A). No significant impacts could be detected either on root length (Fig. 4B) or on leaf surface (Fig. 4D), and the sole *35S::SlFWL2#2* line was negatively affected for hypocotyl length, with a reduction of an average of 20% when compared with the WT (Fig. 4C).

The most noticeable effects were obtained for CRISPR knockout mutants. Two out of three *SlFWL2* mutant lines (namely *CR-Slfwl2#1* and *CR-Slfwl2#2*), and the sole *CR-Slfwl5#3* line displayed a statistically significant reduction in fruit weight (–17%, –42%, and –24%, respectively, on average when compared with the WT) (Fig. 4A). All three *CR-Slfwl4* knockouts lines were affected for root length, with an increase from 15% to 23% on average, when compared with WT plants, while *CR-Slfwl2 and -5* lines were not affected (Fig. 4B; Supplementary Fig. S5). In plants harboring non-functional *SlFWL5* alleles, the length of the hypocotyl in all three *CR-Slfwl5* lines was reduced from 22% to 38% on average, when compared with the WT (Fig. 4C). More remarkably, all three *CR-Slfwl5* lines displayed a marked reduction in leaf surface (from 32% to 47% o average) (Fig. 4D), which suggests that the suppression of *SlFWL5* alters the overall vegetative development of aerial organs, as root development was not affected.

These results suggested that vegetative growth and development in tomato are regulated by SlFWL4 in roots and, more remarkably, by SlFWL5 in leaves and hypocotyls.

### SlFWL5 participates in the control of leaf and hypocotyl growth via cell expansion

**Fig. 5.**
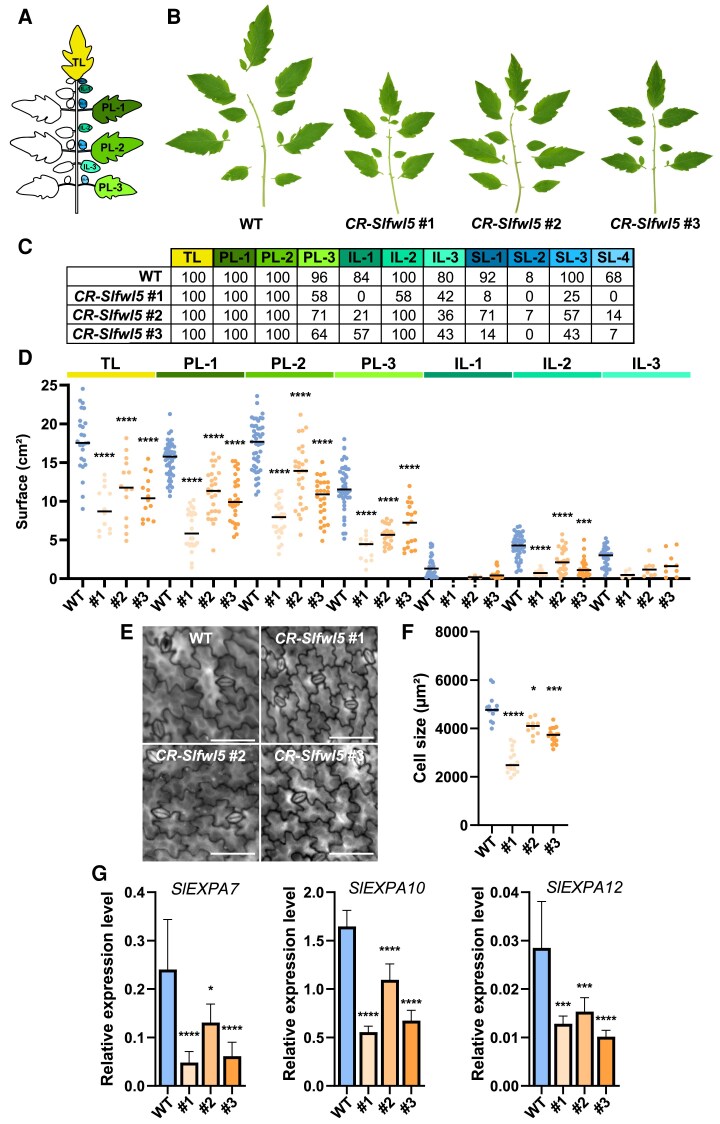
Phenotypic analysis of mature leaves from *CR-Slfwl5* plants compared with those of the WT. (A) Schematic representation of a mature leaf from the WT AC background defining the terminal leaflet (TL), the primary (PL), secondary (SL), and intercalary leaflets (IL) in their sequential order. (B) Silhouettes of the sixth leaf of 8-week-old *CR-Slfwl5* plants. (C) Analysis of leaf composition in *CR-Slfwl5* plants; numbers indicate the percentage of leaves presenting the corresponding leaflet. The color code refers to (A). (D) Determination of the mean TL, PL, and IL surface in WT and *CR-Slfwl5* plants. *n*>10 leaves from four plants per genotype (each dot represents one leaflet surface measurement). Statistical analysis: Kruskal–Wallis test with post-hoc Dunn multiple comparison test. ***P<0.001; ****P<0.0001. (E) Leaf epidermal cells of the WT and the three *CR-Slfwl5* plants. Scale bars=100 µm. (F) Cell size determination in the leaf epidermis of the WT and the three *CR-Slfwl5* plants. Statistical analysis: Kruskal–Wallis test with post-hoc Dunn multiple comparison test. *P<0.05; ***P<0.001; ****P<0.0001. *n*>12 image measurements from leaves 5 and 6 of five independent plants for each line. (G) Expression of *SlEXPA7*, *SlEXPA10*, and *SlEXPA12* in leaves 3–4 of 6-week-old WT and *CR-Slfwl5* plants.

We then proceeded to a fine phenotyping of the leaf morphology in all three *CR-Slfwl5* lines since the overall leaf surface was reduced compared with the WT. The fully expanded compound leaf of the cultivated tomato cultivar AC is made up of a terminal leaflet (TL) and several lateral leaflets attached to the rachis (Fig. 5A). According to the sequential order and position in the rachis, the lateral leaflets can be further categorized into primary (PL), secondary (SL), and intercalary (IL) leaflets.

When compared with WT leaves, the leaf morphology of all three *CR-Slfwl5* lines was affected, as the production of PL-3, IL-1, IL-3, and SLs was greatly reduced (Fig. 5B). The *CR-Slfwl5#1* line displayed systematically the most altered leaf composition with the lowest number of leaflets. Not only was this reduction in the leaflet number responsible for the overall reduction in leaf surface observed in all three *CR-Slfwl5* lines compared with the WT (Fig. 5C), but the reduction in the surface of TL, PL, and IL also contributed to the phenotype (Fig. 5D). This reduction in leaflet surface and overall leaf surface in the three *CR-Slfwl5* lines was due to a reduction in cell size ranging from 17% to 46% on average (Fig. 5E, F). The expression levels of *EXPANSIN A* (*EXPA*) genes used as cell expansion markers (Cosgrove, 2000) were further examined in leaves of 3- of 6-week-old plants of *CR-Slfwl5* lines by RT–qPCR. Among the different *EXPA* genes expressed in tomato leaves (Lu *et al.*, 2016) (Supplementary Fig. S6A), the expression levels of three genes, namely *SlEXPA7*, *-A10*, and *-*A12, were significantly lower in the three mutant lines compared with the WT (Fig. 5G), thus accounting for the alteration of the cell expansion process when the function of SlFWL5 is suppressed.

**Fig. 6.**
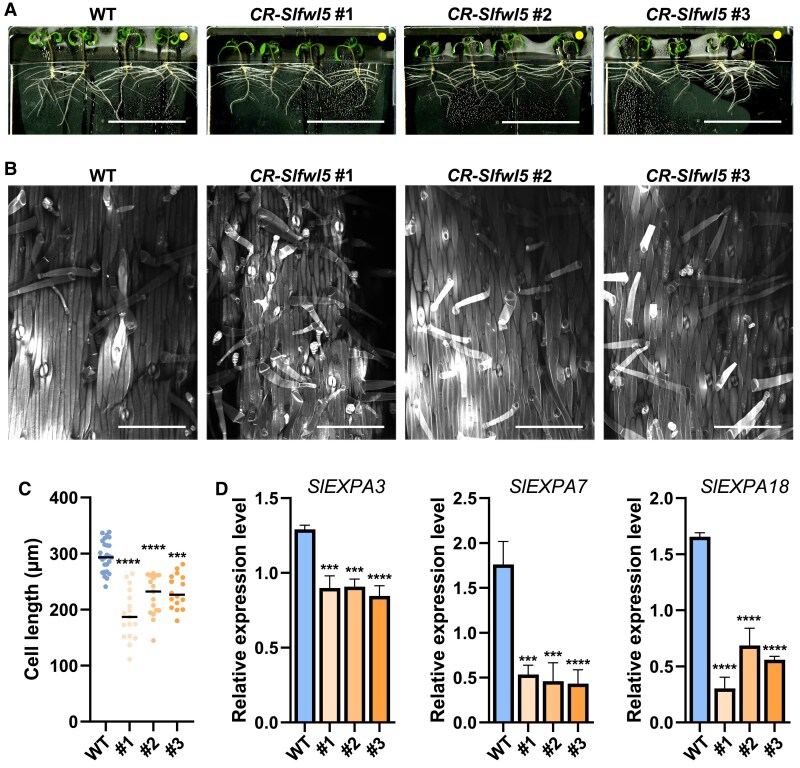
Phenotypic analysis of hypocotyls from *CR-Slfwl5* plants compared with those of the WT. (A) Illustration of hypocotyl length in 2-week-old WT and *CR-Slfwl5* loss-of-function tomato plants. Scale bars=10 cm. (B) Hypocotyl epidermal cells in 10 DAG WT and *CR-Slfwl5* seedlings. Scale bars=200 µm. (C) Cell size determination in the hypocotyl epidermis of WT and *CR-Slfwl5* plants. Statistical analysis: Kruskal–Wallis test with post-hoc Dunn multiple comparison test. ***P<0.001; ****P<0.0001. *n*>12 images measurement from the central part of the hypocotyl of five independent plants for each line. (D) Expression of *SlEXPA3*, *SlEXPA7*, and *SlEXPA18* in hypocotyls of 10 DAG WT and *CR-Slfwl5* seedlings.

Since hypocotyl growth was also affected in the *CR-Slfwl5* lines (Figs 4C, 6A), we next investigated whether the phenotype originated from a defect in cell growth. Clearly, the size of epidermal cells in the hypocotyl of seedlings at 10 days after germination (DAG) was significantly reduced in all *CR-Slfwl5* lines compared with the WT (Fig. 6B), from 21% to 36% on average (Fig. 6C). Here too, *CR-Slfwl5#1* was the most affected line. Similarly to what was observed in leaves, only three *EXPA* genes among those expressed in stems (Lu *et al.*, 2016) (Supplementary Fig. S6B), namely *SlEXPA3*, *-A7*, and *-A18*, were significantly reduced in all *CR-Slfwl5* lines (Fig. 6D). The transcript levels of *SlEXPA7* and *SlEXPA18* displayed the strongest down-regulation.

### SlFWL5 pull-down reveals plasma membrane- and plasmodesmata-related proteins

The elucidation of the functional role of SlFWL5 in leaves and at PDs relied on a deeper characterization at the biochemical level. For this purpose, we performed an immunoprecipitation followed by tandem MS (IP-MS/MS) on *35S::SlFWL5-YFP* leaves to identify interacting protein partners of SlFWL5. This proteomics approach was required to determine the most appropriate leaf developmental stage to harvest, so that the natural interacting protein partners are present in the protein extracts. Hence, the expression level of *SlFWL5* was monitored in the different leaves of 4-week-old plants (Supplementary Fig. S7). Four-week-old plants produced up to eight leaves: from the newly formed leaves, such as leaves 8 and 7 emerging from the meristem, to the oldest fully developed leaves, such as. leaves 2 and 1. The highest expression of *SlFWL5* was found in leaves 6, 5, and 4, when leaf growth is highly sustained by cell expansion. Protein extracts were then prepared from these three different stages and pooled prior to immunoprecipitation.

**Fig. 7.**
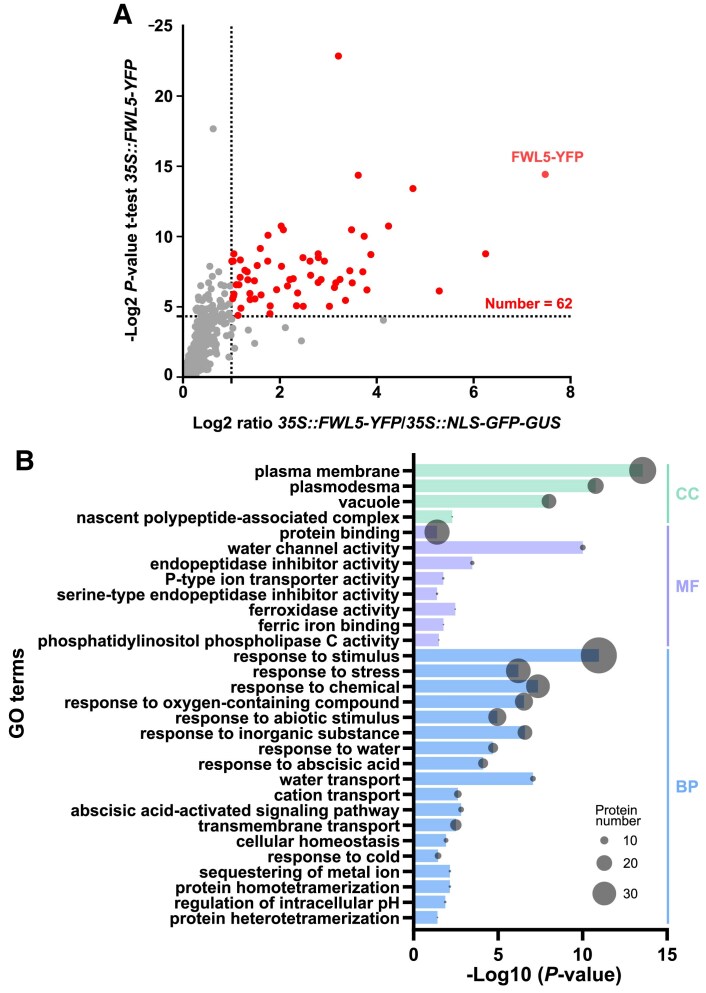
SlFWL5 co-immunoprecipitates with several PM- and PD-localized proteins. (A) Dot plots showing enriched proteins in *35S::SlFWL5-YFP* IP-MS/MS experiments in young leaves. Red dots indicate significantly enriched proteins (based on a Student’s *t*-test with Benjamini–Hochberg correction *P*<0.05 and an enrichment ratio >2). (B) Gene Ontology (GO) enrichment analysis of the identified proteins following SlFWL5 co-immunoprecipitation.

The IP-MS/MS experiment resulted in the identification of 62 proteins that co-immunoprecipitated with SlFWL5, which were significantly enriched in the *35S::SlFWL5-YFP* sample when compared with *35S::NLS-GFP-GUS* used as a control (Fig. 7A; Supplementary Dataset S1). A GO term enrichment analysis for the 62 identified proteins was performed (Fig. 7B), showing that they fall into the three types of GO domains: cellular components (CC), molecular functions (MF), and biological processes (BP). Notably, the CC terms ‘plasma membrane’ and ‘plasmodesmata’ are highly represented. Interestingly, 19 proteins out the 62 identified candidate proteins were found to belong to the FW2.2 co-immunoprecipitation proteome (Beauchet *et al*., 2024), and three of them also crossed with the refined PD proteome from Arabidopsis established by Brault *et al*. (2019), among which was Callose Synthase 10a (SlCalS10a; Solyc03g111570) (Supplementary Table S3). It is also noteworthy that among the identified candidate proteins was a subunit of the Cellulose Synthase complex (SlCesA1; Solyc08g061100), which may relate to cell wall synthesis and cell expansion.

## Discussion

PLAC8 domain-containing proteins in plants belong to the FW2.2-LIKE/CELL NUMBER REGULATOR (FWL/CNR) protein family. Our understanding of the function of FWL/CNR proteins still remains poorly developed, but three distinctive functions have been identified so far (Beauchet *et al*., 2021). These functions relate to (i) calcium uptake and signaling, (ii) metal ion transport and homeostasis, and (iii) organ size determination via the regulation of cell number. These three functions are not exclusive, as they may directly or indirectly influence each other, and examples of mutual actions on plant growth and metal ion transport (Song *et al*., 2015; Xiong *et al*., 2018; Li *et al*., 2019; Gao *et al*., 2020) or plant growth and calcium signaling (Rosa *et al*., 2017) have been reported. It is noteworthy that a function in improving resistance to a plant pathogen, namely *Xanthomonas oryzae*, has been described alongside metal ion transport (Li *et al*., 2019).

So far, functional studies of *FWL/CNR* genes are still scarce in the literature. The first reports were dedicated to the functional characterization of *FW2.2* orthologs in important cereal crops such as maize and rice. In maize, the overexpression of *ZmCNR1* resulted in a reduction in overall plant size and organ size (namely tassel, ear, and leaf size), thus affecting the plant biomass (Guo *et al*., 2010). In rice, T-DNA insertion mutants of *OsFWL3* and *OsFWL5* displayed an increase in the grain weight and/or plant height (Xu *et al*., 2013; Song *et al*., 2015). Similarly, OsFWL1/OsCNR1 (Ruan *et al*., 2020) and OsFWL4 (Gao *et al*., 2020) were shown to act as negative regulators of rice grain width and weight, and tiller number and plant yield, respectively. In addition, the RNAi silencing of *GmFWL1* expression and the deletion of the conserved PLAC8 domain of *GmFWL3* using CRISPR/Cas9 in soybean resulted in a significant reduction in nodule number in response to rhizobial infection, pointing to a critical role for GmFWL1 and GmFWL3 in nodule organogenesis (Libault *et al*., 2010; Cervantes-Pérez *et al*., 2024). In all cases, these studies confirmed a role for certain *FWL/CNR* genes in negatively regulating cell number, thus influencing the organ size and/or development.

Due to the scarcity of data, in the present report we have undertaken a functional analysis of some FWL/CNRs in tomato, so as to enrich our knowledge and to decipher their putative role in plant development.

### The FWL/CNR protein family presents a high diversity in protein characteristics, expression pattern, and subcellular localization in tomato

The *FWL/CNR* gene family in tomato comprises 21 members, showing a large diversity in protein length, ranging from 98 (SlFWL1) to 505 (SlFWL19) amino acids. FWL/CNR proteins share, as a common feature, the presence of the PLAC8 domain made of two conserved motifs: the more or less divergent Cys-rich motif 1 of type CLXXXXCPC or CCXXXXCPC, and the more conserved QXXRELK motif 2 (Fig. 1). Since the CLXXXXCPC motif is present in FW2.2, ZmCNR1, OsFWL1/OsCNR1, and PfCNR1, it would be tempting to address this motif as a signature for FWL/CNR proteins involved in the regulation of organ growth. However, OsFWL4 and OsFWL5, which regulate rice grain weight, harbor the CCXXXXCPC motif. This latter motif has been described as important for conferring the cadmium resistance function (Song *et al*., 2004). Interestingly, OsFWL4 and OsFWL5 both also enhanced cadmium resistance in yeast cells, and OsFWL4 acts as a transporter ensuring the translocation of cadmium from roots to shoots in rice (Song *et al*., 2015; Xiong *et al*., 2018). Therefore, the nature of the PLAC8 Cys-rich motif does not indicate any function with certainty, and this is all the more so since different divergent motifs exist in the tomato FWL/CNR family members. This was particularly obvious for the three SlFWL proteins we characterized functionally in more depth, namely SlFWL2, SlFWL4 and SlFWL5, which harbor a CCXXXXCPC, ALXXXXFPC and AVXXXXLPC motif, respectively (Fig. 1).

Despite clear levels of overexpression (Supplementary Fig. S3), we did not observe any phenotypes in the *35S::SlFWL2*, *35S::SlFWL4*, and *35S::SlFWL5* lines (Fig. 4). This suggests that constitutive overexpression probably fails to reproduce the native spatial and temporal regulation of these *FWL/CNR* genes, or may trigger compensatory mechanisms. On the contrary, the inactivation of only SlFWL4 (*CR-Slfwl4* plants) and SlFWL5 (*CR-Slfwl5* plants) generated marked phenotypes during vegetative development: *CR-Slfwl4* lines produced longer roots (Fig. 4B) and *CR-Slfwl5* lines showed reduced hypocotyl length (Fig. 4C), and a spectacular reduction in leaf size (Figs 4D, 5B). Apart from SlFWL4 which seemed to be preferentially expressed in roots, these phenotypes could not be related to expression of any specific gene: SlFWL2 was found more expressed in cotyledons, and SlFWL5 was expressed in all organs, without any preferential expression in leaves (Supplementary Fig. S1).

In the present study, we investigated the subcellular localization of 11 out of 20 SlFWLs (Fig. 2). A diversity in subcellular localization was observed, as SlFWLs are located according to three distinct patterns: in both the cytosol and the nucleus for SlFWL7 and SlFWL8; at the ER for SlFWL1, -6, -9; -10, -11, and -15; and at the PM for SlFWL2, -4, and -5. Interestingly, a punctate localization in the PM was observed for SlFWL2 and -4, which may be consistent with a membrane microdomain localization, as demonstrated for GmFWL1 and GmFWL3 in soybean nodules (Qiao *et al*., 2017; Cervantes-Pérez *et al*., 2024). Therefore, this diversity in subcellular localization might be indicative of a diversity in protein function.

With the exception of SlFWL7 and SlFWL8, all the tested SlFWLs were thus found targeted to membranous compartments, most probably in connection with the presence of the hydrophobic PLAC8 domain or TM domains in the case of SlFWL15. From the original structural analysis of AtPCR1 (Song *et al*., 2004), it has long been accepted, and widely and systematically reported in the literature, that the PLAC8 domain in plant FWL/CNRs is composed of two hydrophobic segments (including the CLXXXXCPC or CCXXXXCPC motif), predicted to form TM domains (for a review, see Beauchet *et al*., 2021). However, the use of currently available tools for TM topology prediction revealed that SlFWL2, SlFWL4, and SlFWL5 may not cross the PM in the absence of TM domains, but are likely to be anchored in the outer leaflet of the PM via their PLAC8 hydrophobic domain (Supplementary Fig. S2), Such a topology would thus be very similar to that demonstrated experimentally for FW2.2 (Beauchet *et al*., 2024).

### SlFWL5 regulates leaf size and hypocotyl growth in tomato via cell expansion

Unexpectedly, we could demonstrate that the leaf and hypocotyl growth phenotypes in *CR-Slfwl5* lines are not due to impaired cell divisions, as would be expected for a member of the CELL NUMBER REGULATOR protein family, but from an impairment in cell expansion, as cell size in all three lines was greatly reduced (Figs 5E, F, 6B, C). Interestingly, this reduction in cell size was accompanied by a significant down-regulation of some leaf- and hypocotyl-expressed *EXPA* genes (Figs 5G, 6D). The contribution of the *EXPANSIN* gene expression in modulating leaf growth (Cho and Cosgrove, 2000; Goh *et al*., 2012) and hypocotyl growth (Caderas *et al*., 2000) is a long-standing observation. For leaf growth in particular, it is part of the complex molecular and hormonal network regulating leaf size (Gonzalez *et al*., 2012).

Not all *EXPA* genes responded in the same way to *SlFWL5* loss of function. Indeed, among the genes specifically expressed in these two organs (Lu *et al*., 2016), only *SlEXPA7*, *-10*, and *-12*, and *SlEXPA3*, *-7*, and *-18*, were down-regulated in leaf and hypocotyl, respectively (Figs 5G, 6D; Supplementary Fig. S6). The origin of this discrepancy is not known. It can be related to the specific function of EXPANSINS or the timing and localization of *EXPA* gene expression. For instance, *SlEXPA2* is the major gene expressed in stems and hypocotyls, but its expression is maximal at the top of the hypocotyl, corresponding to the zone of rapid elongation, and very low in the middle part of the hypocotyl where cells have already expanded (Caderas *et al*., 2000). Whether this discrepancy may relate to any modification in the hormonal regulation of *EXPA* genes following SlFWL5 loss of function is an intriguing subject for investigation. It was shown that *SlEXPA3*, *-7*, *-12*, and *-18* were all positively regulated by auxin, while *SlEXPA10* was positively regulated by gibberellin (GA) (Lu *et al*., 2016), two major hormones involved in the stimulation of cell elongation during plant growth.

The inactivation of *SlFWL5* not only affected leaf growth, but it also altered leaf morphology into simpler leaves, displaying a reduced number of the third PL, and of SLs,and ILs (Fig. 5C, D). This phenotype is fully relevant to hormonal control of compound leaf morphogenesis and differentiation in tomato (Bar and Ori, 2015). Indeed, GA negatively regulates leaf complexity in tomato, as increased GA levels or GA signaling accelerate leaf maturation and induce simpler leaves than in the WT (Jasinski *et al*., 2008; Yanai *et al*., 2011). In addition, GA and cytokinin (CK) present a mutual antagonistic interaction during tomato leaf development (Fleishon *et al*., 2011). How to relate the molecular origin of the leaf phenotype in *CR-Slfwl5* plants and phytohormone signaling remains to be established, but the following step in the functional characterization of SlFWL5 may provide new lines of research.

### SlFWL5 is enriched at plasmodesmata, within a protein complex composed of plasmodesmata- and plasma membrane-specific proteins

We have shown that SlFWL5 is enriched at PDs (Fig. 3), where it probably participates in a protein complex composed of PM- and PD-specific proteins (Fig. 7). Interestingly, a significant number of candidate proteins that co-immunoprecipitated with SlFWL5 were found enriched at PDs. This finding suggests that SlFWL5 may have a function in cell-to-cell communication mechanisms for the control of leaf and hypocotyl growth. SlFWL5 co-immunoprecipitates with CalS10a (Solyc03g111370), which contributes to callose homeostasis at the cell plate and at PDs, thereby regulating cytokinesis and the symplastic molecular exchanges between neighboring cells via the permeability of PDs (Saatian *et al*., 2018). CalS10 is required for normal plant development, as silencing of *CalS10* results in retarded growth: plants display dwarfism, with a smaller stem and smaller leaves (Töller *et al*., 2008), a phenotype originating from either impaired cell division or cell expansion. *CR-Slfwl5* loss-of-function plants displayed a similar phenotype, namely a reduced hypocotyl length and leaf surface, due to impaired cell expansion. However, our data indicate that SlFWL5 does not directly control callose deposition dynamics via regulating the CalS activity, thereby modifying the PD aperture. Indeed, according to our immunolocalization assays, the absence of a functional SlFWL5 was not associated with measurable differences in callose deposition in leaves (Supplementary Fig. S8). Therefore, the mode of action of SlFWL5 at PDs appears quite different from that of FW2.2 (Beauchet *et al*., 2024), and the putative interaction of SlFWL5 with CalS10 may only reflect its protein environment at PDs.

Interestingly, SlCesA1, a subunit of the Cellulose Synthase complex, was identified among the candidate proteins that co-immunoprecipitate with SlFWL5. Cellulose is the most abundant β-glucan polysaccharide of the plant cell wall, which self-assembles as microfibrils to strengthen the cell wall and contribute to the direction of cell growth during the cell expansion process (Schneider *et al*., 2016). Both cellulose and callose are synthesized at the PM by the large respective synthase complexes, cellulose synthases and callose synthases, and the formation of cellulose–callose networks has been demonstrated, especially as a layered cellulose–callose architecture in cell walls of epidermal leaf cells (Falter *et al*., 2015). Whether the SlFWL5 function in controlling leaf growth requires a regulatory role on cellulose synthases during cell expansion remains an exciting avenue for future investigation.

Unfortunately, we were unable to provide any direct experimental evidence for the physical interaction between SlFWL5 and CalS10 or SlCesA1, identified as putative interactors from our IP-MS/MS proteomics assays (Fig. 7; Supplementary Table S4). First, the very large size and multi-TM domain nature of CalS and CesA make them extremely challenging to clone and express in heterologous system. Second, both CalS and CesA proteins possess N- and C-terminal ends that face the cytosol (Verma and Hong, 2001; Kumar and Turner, 2015), whereas SlFWL5 displays both N- and C-terminal regions in the apoplast (Supplementary Fig. S2), like its counterpart FW2.2 (Beauchet *et al*., 2024). Therefore, this differential orientation precludes proper reconstitution of split-ubiquitin fragments or fluorescent tags, even if fused, resulting in false negatives or structural artifacts when using classical interaction methods, such as yeast two-hybrid or split-ubiquitin. Moreover, PDs and plant-specific membrane microdomains have unique lipid composition and regulatory environments that do not exist in yeast. Consequently, assays in yeast may not reflect biologically relevant interactions in plants. While *in planta* approaches (such as FRET-FLIM/BiFC) might appear suitable, the opposite orientation of N- and C-termini and thus this difference in protein topology prevent reliable reconstitution, and compromise the demonstration of a direct structural interaction between SlFWL5 and CalS10 or SlCesA1.

As key elements of the cell-to-cell communication machinery, PDs are implicated in processes guaranteeing the collaborative function of the cells, and developmental and patterning events (Petit *et al*., 2020), enabling the intercellular trafficking of different mobile signaling molecules, such as small RNAs, metabolites, hormones, and transcription factors (Wu and Gallagher, 2012; Brunkard *et al*., 2013). Being localized at PDs, SlFWL5 may have a regulatory role in PD activity. Hence, we can hypothesize that SlFWL5 function is to regulate the cell-to-cell movement of molecules/signals promoting leaf cell expansion and leaflet initiation. Obviously, the identification of such signaling molecules belonging to transcriptional networks involved in pathways affecting cell growth (Davière and Achard, 2013) and leaf growth (Gonzalez *et al*., 2012; Bar and Ori, 2014) would represent a giant leap in understanding the SlFWL5 functional role at PDs.

In conclusion, we are far from deciphering the functional complexity of the FWL/CNR protein family during plant and organ development. Unexpectedly, we have been able to demonstrate that SlFWL5 contributes to the regulation of cell expansion during organ growth, which has never been reported before for a member of the CELL NUMBER REGULATOR protein family. Therefore, this study may lead us to revise our perception of the FWL/CNR function in regulating organ size. Whatever the mechanism involved, cell division or cell expansion control, what may really matter is the potential function in cell-to-cell communication so as to regulate organ growth.

## Supplementary Material

eraf444_Supplementary_Data

## Data Availability

The Mass Spectrometry Proteomics data underlying this article have been deposited to the ProteomeXchange Consortium via the PRIDE (Perez-Riverol *et al*., 2022) partner repository with the dataset identifier PXD061438.

## Supplementary data

The following supplementary data are available at *JXB* online.

Table S1. List of primers used for constructs and RT–qPCR analysis.

Table S2. List of Gateway vectors used for constructs.

Table S3. List of candidate proteins co-immunoprecipitating with SlFWL5.

Fig. S1. *SlFWL* gene expression analysis in different organs of tomato plants and during fruit development.

Fig. S2. Structure and membrane insertion predictions of SlFWL2, SlFWL4, and SlFWL5 compared with those of FW2.2.

Fig. S3. RT–qPCR analysis of *SlFWL2*, *SlFWL4*, and *SlFWL5* expression in leaves of their respective overexpressing lines.

Fig. S4. CRISPR/Cas9-induced mutations producing truncated versions of SlFWL2, SlFWL4, and SlFWL5.

Fig. S5. Illustration of root phenotype in WT and *CR-Slfwl4* loss-of-function tomato plants.

Fig. S6. Expression levels of *EXPANSIN A* genes in leaves and hypocotyls from *CR-Slfwl5* plants compared with those of the WT.

Fig. S7. RT–qPCR analysis of SlFWL5 expression in the leaves of 4-week-old plants.

Fig. S8. The loss of function of SlFWL5 does not alter callose deposition in leaves.

Dataset S1. Raw datasets from IP-MS/MS following SlFWL5–YFP co-immunoprecipitation.
