# Supplementary material for: The CELL NUMBER REGULATOR SlFWL5 protein regulates aerial vegetative growth in tomato, by promoting cell expansion
Source: J Exp Bot. 2025 Oct 15;77(2):543–58. doi: 10.1093/jxb/eraf444 (PMC12794220; doi:10.1093/jxb/eraf444)
Supplement: eraf444_Supplementary_Data [file eraf444_supplementary_data.zip › eraf444 Supplemental Figures S1 to S8.pdf]

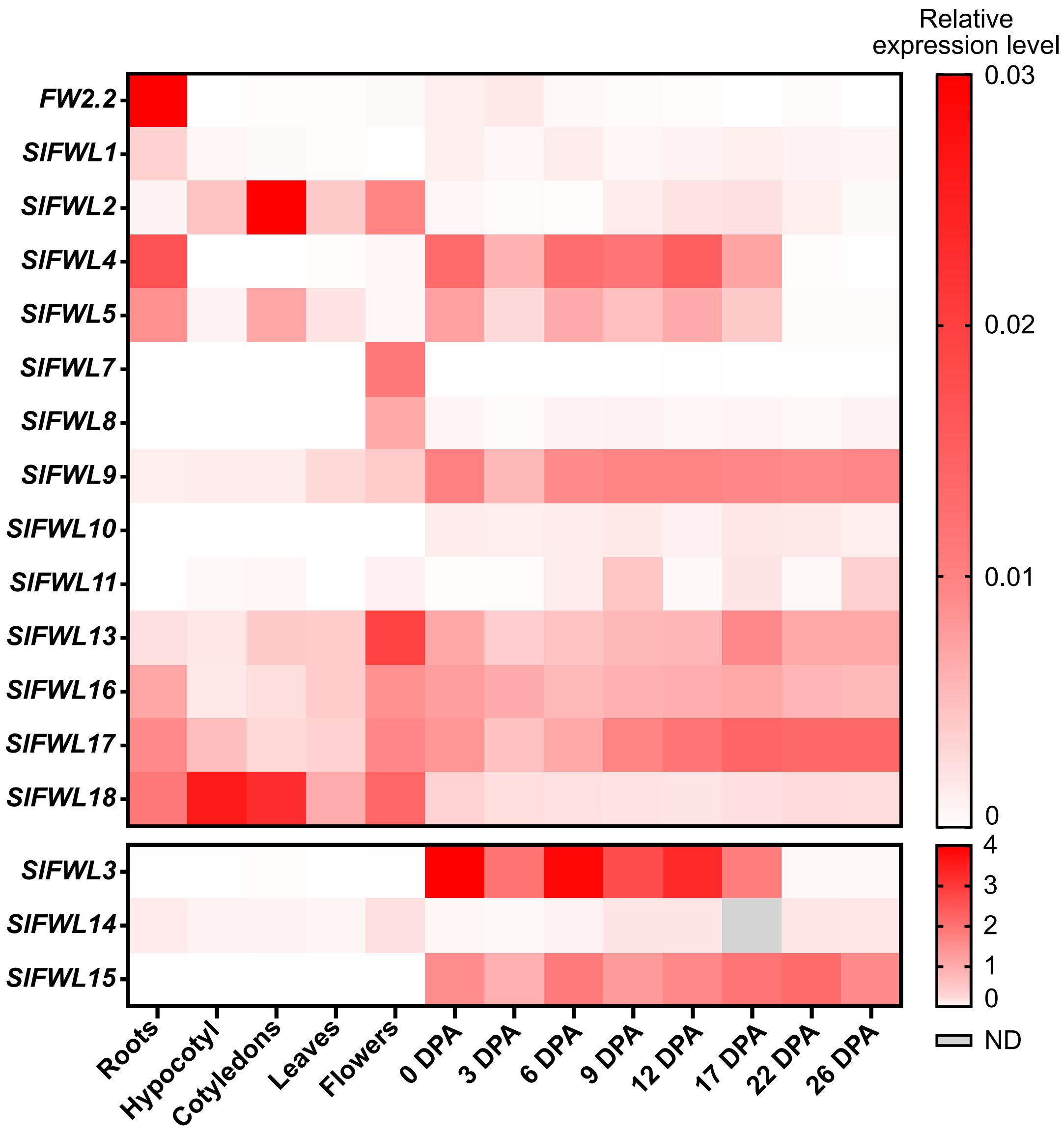

Fig. S1. *SlFWLs* expression analysis in different organs of tomato plants and during fruit development. ND, not determined.

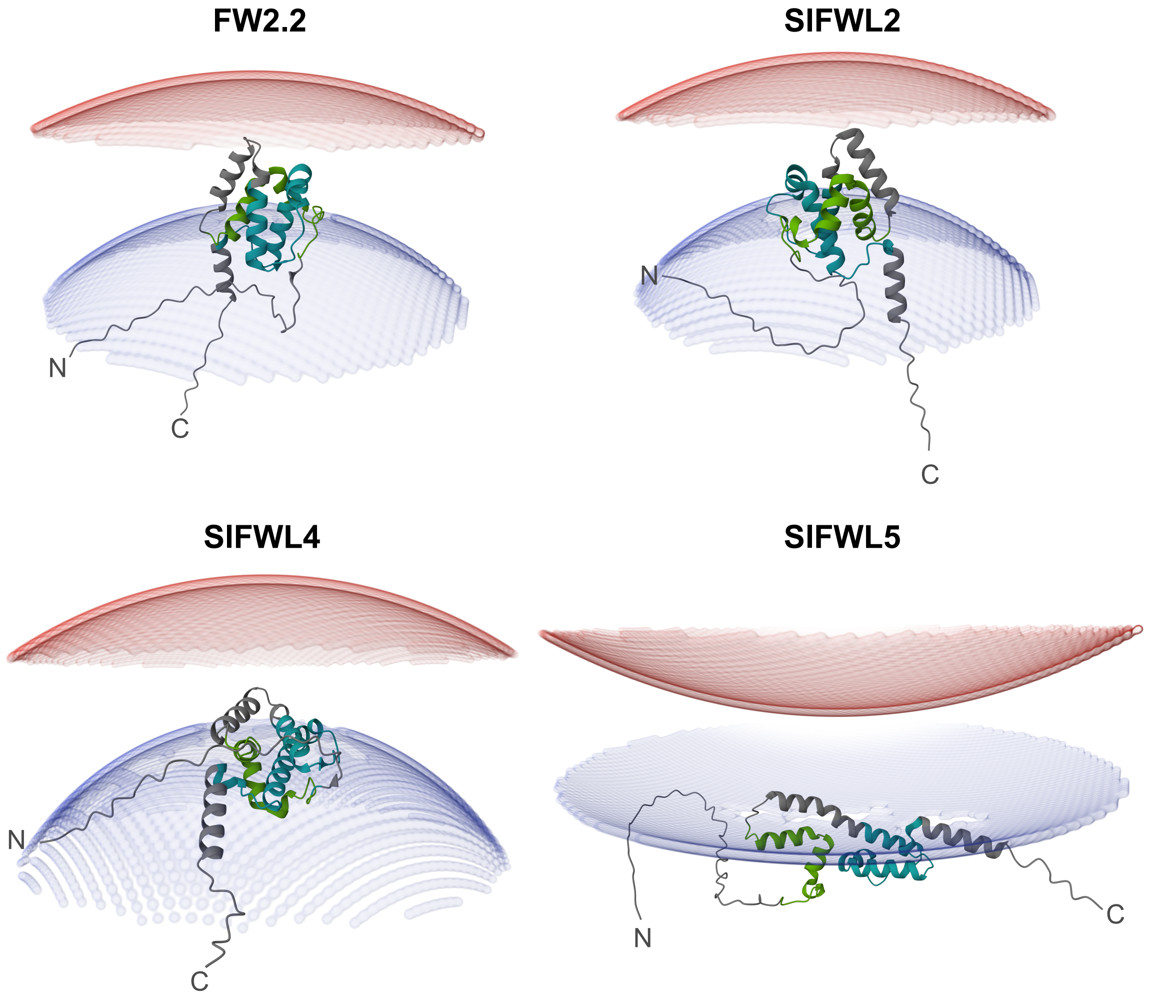

Fig. S2. Structure and membrane insertion predictions of SlFWL2, SlFWL4 and SlFWL5 compared to that of FW2.2, using Colabfold (Mirdita *et al*., 2022) and the PPM 3.0 Web Server (https://opm.phar.umich.edu/ppm_ server3_cgopm) (Lomize *et al*., 2022).

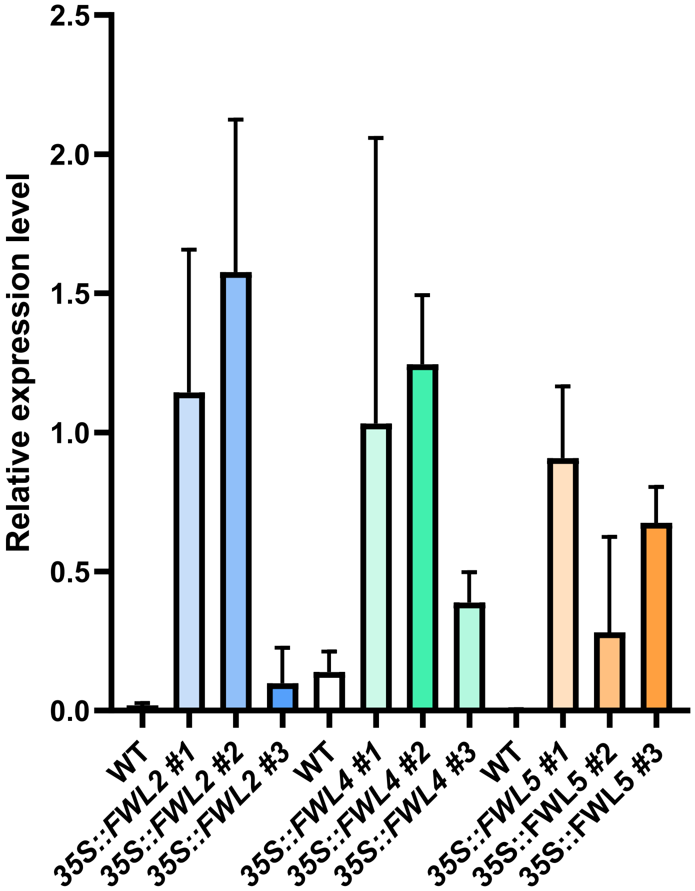

Fig. S3. RT-qPCR analysis of *SlFWL2*, *SlFWL4* and *SlFWL5* expression in leaves of their respective overexpressing lines.

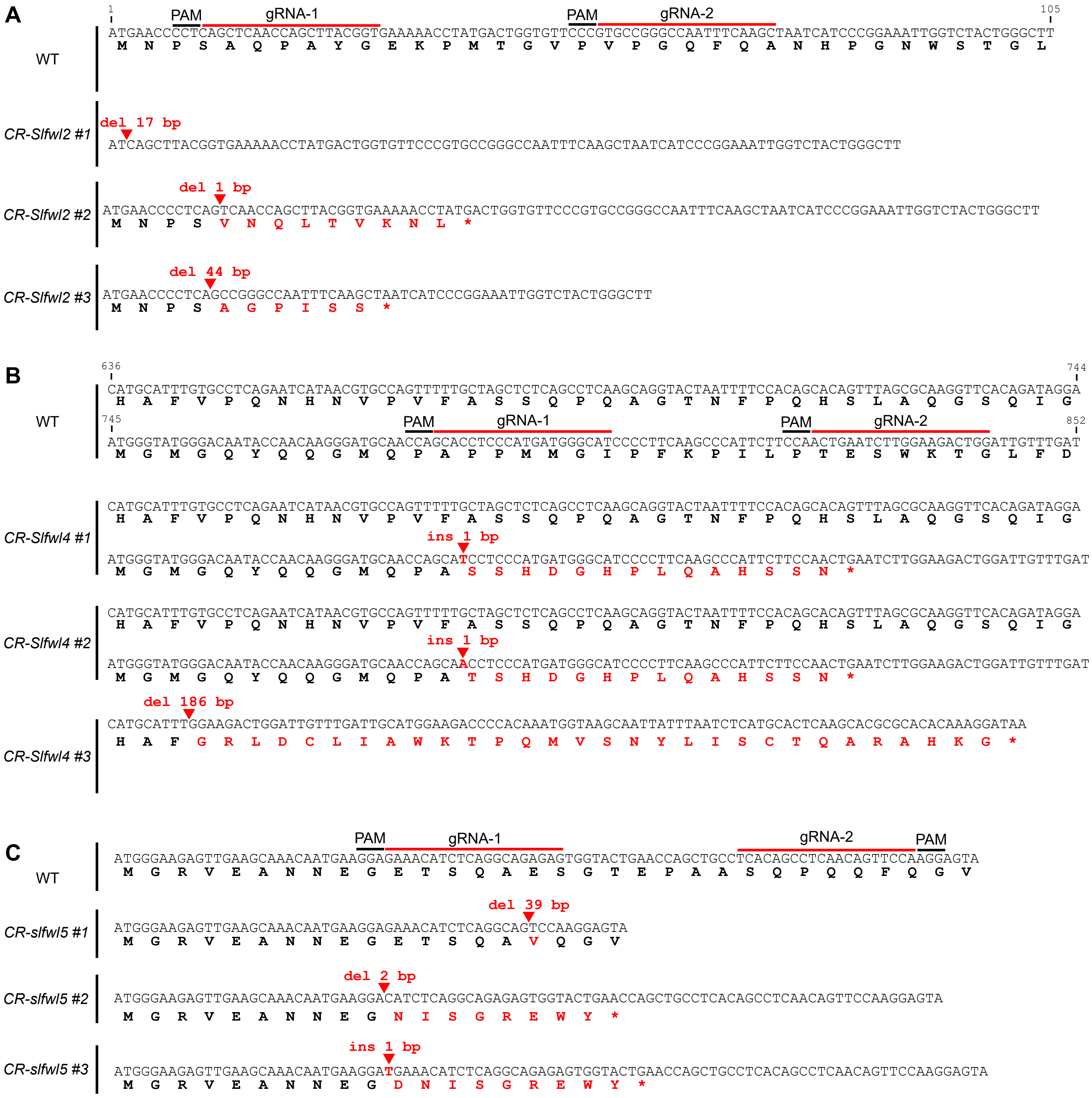

Fig. S4. CRISPR/Cas9-induced mutations producing truncated versions of (**A**) SlFWL2, (**B**) SlFWL4 and (**C**) SlFWL5. The localization and type of the induced mutations is indicated by a red arrow. The corresponding protein sequence is indicated below the nucleotidic sequence; difference in the amino-acid sequences are indicated in red.

WT | *CR-Slfwl4#1* | *CR-Slfwl4#2* | *CR-Slfwl4#3*

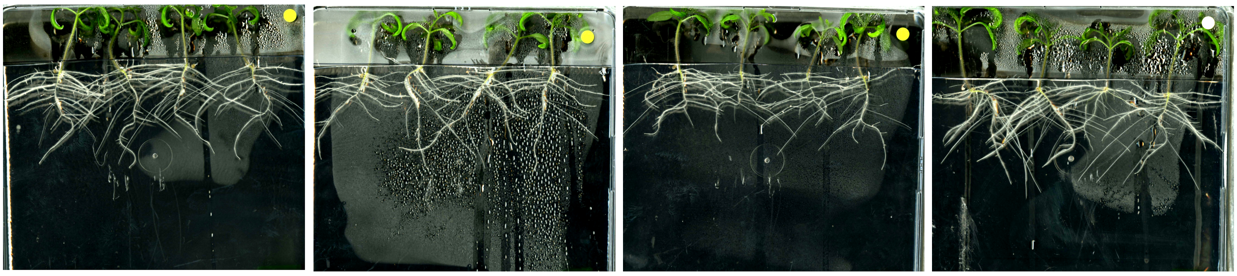

Fig. S5. Illustration of root phenotype in WT and *CR-Slfwl4* loss-of-function tomato plants.

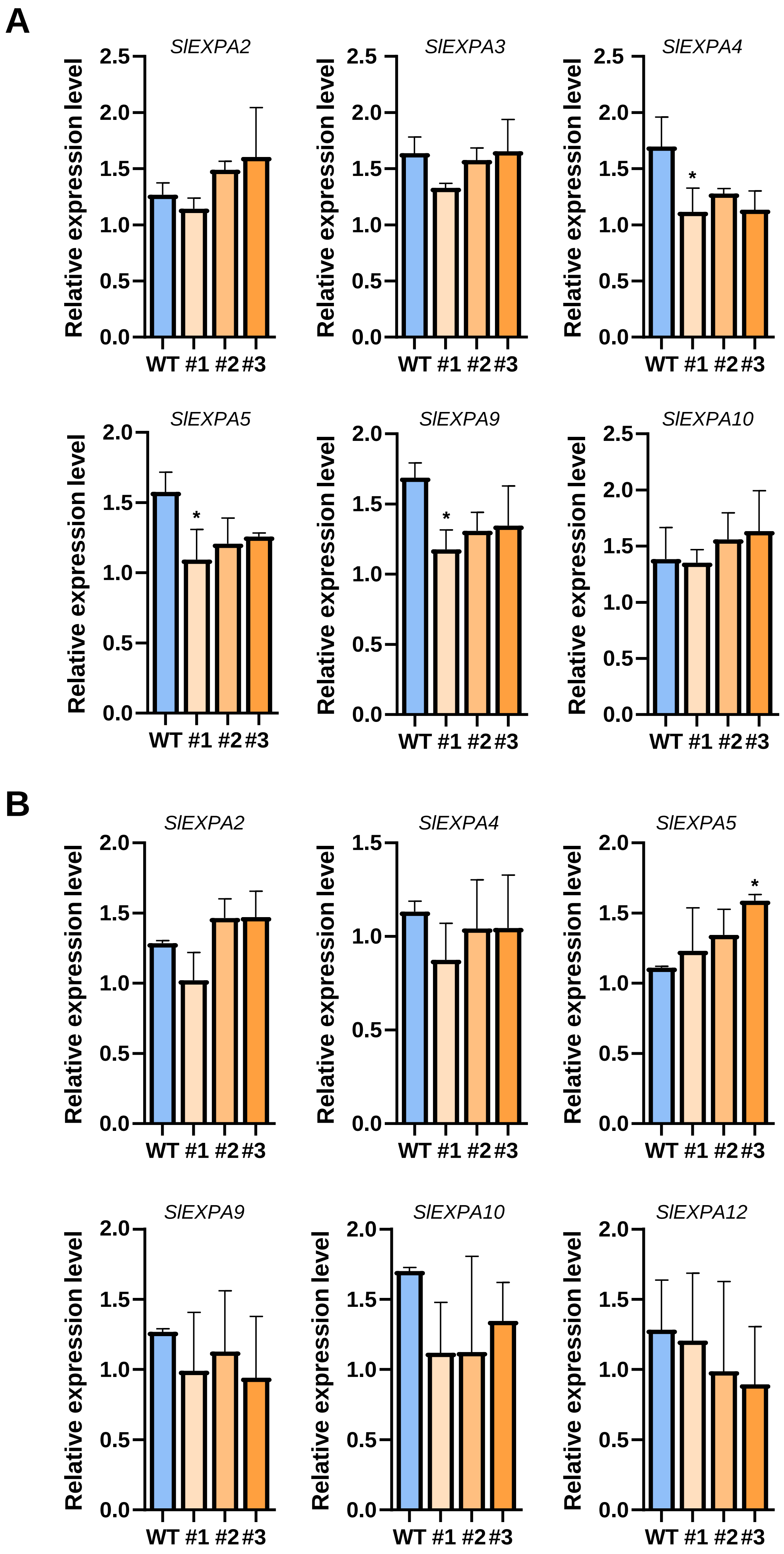

A
SlEXPA2
SlEXPA3
SlEXPA4
SlEXPA5
SlEXPA9
SlEXPA10
B
SlEXPA2
SlEXPA4
SlEXPA5
SlEXPA9
SlEXPA10
SlEXPA12
Relative expression level
WT #1 #2 #3
*

Fig. S6. Expression levels of leaf-specific (A) and hypocotyl-specific (B) *EXPANSIN A* genes in *CR-Slfwl5* plants compared to WT. (A) RT-qPCR expression analysis of *SlEXPA2*, *-3*, *-4*, *-5*, *-9* and *-18* in leaves 3-4 of six-week old WT and *CR-Slfwl5* plants. (B) RT-qPCR expression analysis of *SlEXPA2*, *-4*, *-5*, *-9*, *-10* and *-12* in hypocotyls of 10 DAG WT and *CR-Slfwl5* seedlings.

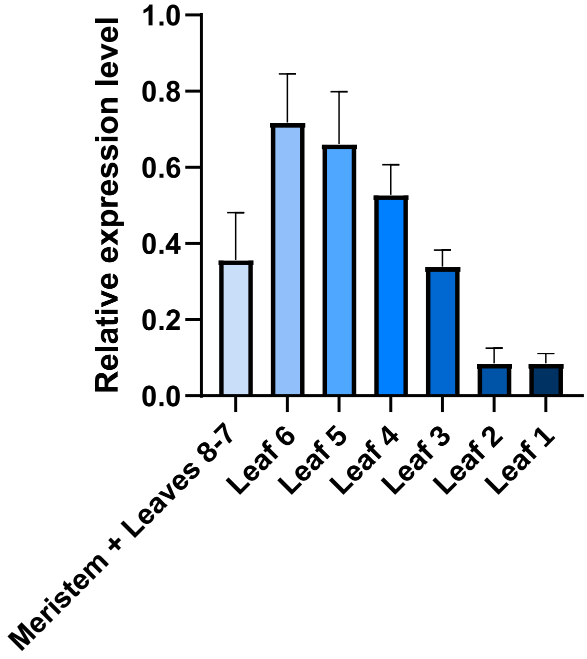

Fig. S7. RT-qPCR analysis of *SlFWL5* expression in the leaves of 4-week old plants.

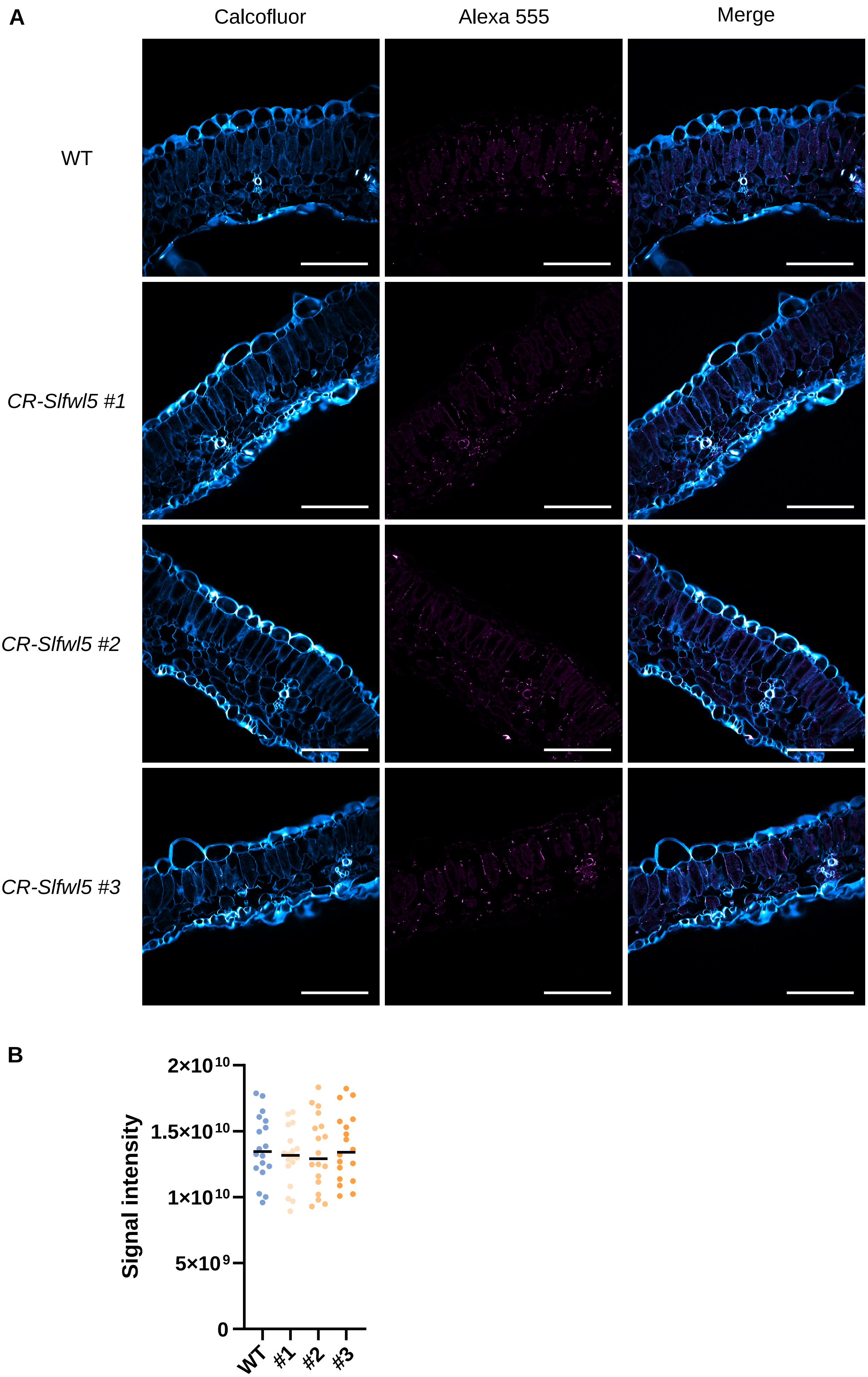

Fig. S8. FWL5 loss-of-function does not alter callose deposition in leaves. (A) Immunolabeling of callose in leaves of WT and *CR-Slfwl5* plants. Scale bar = 100 µm. (B) Quantification of callose deposition in WT and *CR-Slfwl5* lines. The signal intensity for callose deposition is integrated to the pixel surface measured. n = 18 measurements on 3 leaflets from 3 plants.
